# Supplementary material for: Bacterial but Not Fungal Rhizosphere Community Composition Differ among Perennial Grass Ecotypes under Abiotic Environmental Stress
Source: Microbiol Spectr. 2022 Apr 20;10(3):e02391-21. doi: 10.1128/spectrum.02391-21 (PMC9241903; doi:10.1128/spectrum.02391-21)
Supplement: SUPPLEMENTAL FILE 1 — Supplemental material. Download spectrum.02391-21-s001.pdf, PDF file, 1 MB [file spectrum.02391-21-s001.pdf]

Supplementary Files:

Supplementary Table S1: Raw sequence analysis by QIIME 2 Version 2019.7. The number of counts of bacteria and fungi initially obtained, and the counts that were considered after primer trimming and DADA2 quality control per sample.

Bacterial count

Sample_name	Ecotype	original counts	counts after primer trim	counts after DADA2
D-REL-R4	Dry	10972	10398	6269
D-SAL-R3	Dry	11592	10399	6598
D-WEB-R10	Dry	11773	11138	6862
D-REL-R6	Dry	12847	11979	7228
D-WEB-R8	Dry	12209	11511	7310
D-CDB-R5	Dry	12698	11903	7360
D-SAL-R4	Dry	12647	11985	7471
D-CDB-R8	Dry	13417	12662	7837
D-SAL-R1	Dry	11549	10669	8025
D-REL-R7	Dry	13222	12588	8300
D-SAL-R9	Dry	13752	13061	8328
D-SAL-R5	Dry	14140	13286	8589
D-SAL-R6	Dry	14744	13912	8670
D-CDB-R7	Dry	14360	13640	8837
D-WEB-R7	Dry	14148	13447	9156
D-WEB-R6	Dry	14933	13972	9436
D-SAL-R7	Dry	17282	16312	10661
D-REL-R3	Dry	20675	19019	13790
D-WEB-R1	Dry	20428	18618	14027
D-CDB-R6	Dry	22670	21335	14434
D-CDB-R2	Dry	21683	19959	14465
D-WEB-R3	Dry	21466	19910	14756
D-CDB-R9	Dry	23101	22069	15100
D-SAL-R2	Dry	23511	21822	15141
D-REL-R2	Dry	22073	20169	15921
D-SAL-R8	Dry	25259	23997	17179
D-CDB-R1	Dry	23421	21650	17316
D-REL-R8	Dry	25093	23899	17553
D-REL-R9	Dry	26309	24953	17816
D-CDB-R4	Dry	23713	22683	18024
D-WEB-R9	Dry	25071	23791	18351
D-WEB-R5	Dry	24332	23125	18450
D-REL-R10	Dry	26109	24848	18583
M-TAL-R6	Mesic	9022	8428	4485
M-TOW-R7	Mesic	11873	11321	7213
M-KON-R2	Mesic	13312	12714	8224
M-TAL-R10	Mesic	13898	13250	8245
M-TOW-R8	Mesic	13050	12426	8272
M-TOW-R2	Mesic	13180	12494	8378
M-KON-R1	Mesic	12978	12210	8387
M-TOW-R6	Mesic	13081	12444	8537
M-KON-R6	Mesic	14199	13449	8933
M-TOW-R4	Mesic	15337	14631	9051
M-TAL-R1	Mesic	14802	14142	9215
M-KON-R7	Mesic	14031	13359	9288
M-TOW-R1	Mesic	14277	13535	9298
M-TAL-R3	Mesic	14810	14067	9312
M-TOW-R3	Mesic	15320	14565	9428
M-TAL-R5	Mesic	15395	14638	9671
M-KON-R8	Mesic	15365	14618	9753
M-TAL-R4	Mesic	15225	14536	9815
M-KON-R4	Mesic	15453	14716	9848
M-TAL-R8	Mesic	16171	15384	10457
M-TOW-R9	Mesic	16391	15631	10492
M-TAL-R2	Mesic	15815	15067	10766
M-KON-R9	Mesic	17504	16675	11139
M-TAL-R9	Mesic	18097	17336	11877
M-KON-R10	Mesic	19641	18736	12665
M-TAL-R7	Mesic	21302	20230	15068
M-TOW-R10	Mesic	23697	22597	16345
M-TOW-R5	Mesic	23503	22658	16487
M-KON-R3	Mesic	25982	24934	18118
M-KON-R5	Mesic	26087	25105	19042
W-12MI-R8	Wet	10821	10194	6139
W-DES-R10	Wet	11135	10555	6671
W-DES-R8	Wet	11740	11118	7149
W-WAL-R4	Wet	12482	11616	7324
W-DES-R5	Wet	12955	12111	7403
W-12MI-R6	Wet	13453	12824	8023
W-WAL-R6	Wet	13294	12634	8282
W-DES-R9	Wet	14178	13517	8751
W-DES-R6	Wet	15753	14999	9623
W-FUL-R9	Wet	15615	14772	9750
W-12MI-R3	Wet	16116	14977	9951
W-FUL-R6	Wet	16018	15114	12194
W-12MI-R2	Wet	19395	17955	12904
W-WAL-R2	Wet	22183	20626	14386
W-WAL--R1	Wet	22639	20782	14448
W-FUL-R2	Wet	22134	20631	15157
W-DES-R3	Wet	22992	21450	15255
W-12MI-R1	Wet	21668	19837	15278
W-12MI-R10	Wet	22342	21263	15746
W-FUL-R5	Wet	22001	20746	15820
W-FUL-R4	Wet	22058	20705	15866
W-FUL-R1	Wet	22078	20192	15965
W-FUL-R3	Wet	23181	21736	16744
W-WAL-R9	Wet	24920	23780	16872
W-WAL-R8	Wet	24796	23505	17068
W-FUL-R10	Wet	24250	22978	17106
W-WAL-R10	Wet	24766	23524	17111
W-DES-R7	Wet	24881	23605	17406
W-FUL-R7	Wet	26809	25473	18265
W-WAL-R3	Wet	25988	24383	18548
W-WAL-R7	Wet	27202	25936	19422
W-12MI-R7	Wet	29578	28088	19921

Fungal Count				
sample_name	Ecotype	original counts	counts after primer trim	counts after dada2
D-CDB-R1	Dry	50767	50127	32329
D-CDB-R2	Dry	48349	47773	39843
D-CDB-R4	Dry	32022	31367	19595
D-CDB-R5	Dry	41374	40888	25981
D-CDB-R6	Dry	41986	41206	26196
D-CDB-R7	Dry	42382	41620	25928
D-CDB-R8	Dry	38498	37888	22223
D-CDB-R9	Dry	37780	37315	21888
D-REL-R10	Dry	39202	38632	27740
D-REL-R2	Dry	36600	36156	25073
D-REL-R3	Dry	38352	37747	26858
D-REL-R4	Dry	18223	17896	10258
D-REL-R6	Dry	37590	37011	19298
D-REL-R7	Dry	45471	44828	24869
D-REL-R8	Dry	31599	31177	17666
D-REL-R9	Dry	33760	33245	18713
D-SAL-R1	Dry	43221	42674	29101
D-SAL-R2	Dry	31763	31180	22140
D-SAL-R3	Dry	38022	37344	23073
D-SAL-R4	Dry	35964	35411	22682
D-SAL-R5	Dry	44976	44432	26021
D-SAL-R6	Dry	39616	39123	24554
D-SAL-R7	Dry	43540	42834	26815
D-SAL-R8	Dry	30916	30500	17736
D-SAL-R9	Dry	36626	36060	18459
D-WEB-R1	Dry	48767	48094	33764
D-WEB-R10	Dry	35153	34541	20842
D-WEB-R3	Dry	38464	37813	24411
D-WEB-R5	Dry	40540	39790	26860
D-WEB-R6	Dry	55599	54960	32031
D-WEB-R7	Dry	36929	36427	23535
D-WEB-R8	Dry	25445	24995	15373
D-WEB-R9	Dry	19740	19474	11474
M-KON-R1	Mesic	29226	28747	16032
M-KON-R10	Mesic	38833	38400	24812
M-KON-R2	Mesic	34528	33844	19701
M-KON-R3	Mesic	30231	29585	21337
M-KON-R4	Mesic	38676	38001	22780
M-KON-R5	Mesic	34544	33956	23158
M-KON-R6	Mesic	37079	36564	15243
M-KON-R7	Mesic	30456	30023	15886
M-KON-R8	Mesic	40074	39535	23992
M-KON-R9	Mesic	33074	32645	14955
M-TAL-R1	Mesic	36170	35624	26178
M-TAL-R10	Mesic	36506	35907	22582
M-TAL-R2	Mesic	43654	42955	25681
M-TAL-R3	Mesic	35583	34986	20020
M-TAL-R4	Mesic	37304	36632	20500
M-TAL-R5	Mesic	39471	38730	26305
M-TAL-R6	Mesic	36747	36213	20033
M-TAL-R7	Mesic	40414	39840	24998
M-TAL-R8	Mesic	48403	47875	29400
M-TAL-R9	Mesic	42194	41675	26093
M-TOW-R1	Mesic	24381	23971	10695
M-TOW-R10	Mesic	46508	45940	14334
M-TOW-R2	Mesic	23479	23050	10758
M-TOW-R3	Mesic	38444	37801	23121
M-TOW-R4	Mesic	44884	44038	28295
M-TOW-R5	Mesic	32945	32316	19710
M-TOW-R6	Mesic	45693	45102	28776
M-TOW-R7	Mesic	38471	37975	24552
M-TOW-R8	Mesic	47835	47218	33177
M-TOW-R9	Mesic	39433	38925	22062
W-12MI-R1	Wet	38745	38188	24038
W-12MI-R10	Wet	32354	31855	19406
W-12MI-R2	Wet	43136	42446	27451
W-12MI-R3	Wet	28722	28162	16152
W-12MI-R6	Wet	52503	51811	27664
W-12MI-R7	Wet	46031	45381	27701
W-12MI-R8	Wet	41291	40582	27460
W-DES-R10	Wet	32931	32292	18958
W-DES-R3	Wet	33802	33157	19660
W-DES-R5	Wet	41049	40475	25579
W-DES-R6	Wet	51720	51075	34677
W-DES-R7	Wet	34733	34185	26209
W-DES-R8	Wet	30609	30047	18451
W-DES-R9	Wet	40561	39906	23154
W-FUL-R1	Wet	46410	45832	27964
W-FUL-R10	Wet	33015	32635	21928
W-FUL-R2	Wet	38214	37560	26880
W-FUL-R3	Wet	43075	42399	26886
W-FUL-R4	Wet	36184	35438	24071
W-FUL-R5	Wet	41053	40343	25348
W-FUL-R6	Wet	39995	39377	17861
W-FUL-R7	Wet	35861	35303	21777
W-FUL-R9	Wet	24307	23936	12893
W-WAL--R1	Wet	43279	42747	32477
W-WAL-R10	Wet	25190	24837	12735
W-WAL-R2	Wet	39210	38542	22248
W-WAL-R3	Wet	30834	30307	20630
W-WAL-R4	Wet	40100	39601	25528
W-WAL-R6	Wet	49971	49324	29880
W-WAL-R7	Wet	21773	21449	14574
W-WAL-R8	Wet	29567	29135	15406
W-WAL-R9	Wet	19835	19574	10451

Supplementary Table S2: Post-hoc SIMPER analyses and relative abundance of the major bacterial phyla indicate the bacterial and fungal phyla that contributed to major similarities and dissimilarities among the dry, mesic, and wet ecotypes.

Post-hoc SIMPER Analysis (bacteria)

Group Dry	
**Species**	**Contribution (%)**
Proteobacteria	20.98
Actinobacteria	17.06
Acidobacteria	12.6
Chloroflexi	7.52
Bacteroidetes	7.01
Verrucomicrobia	6.36

Group Mesic	
**Species**	**Contribution (%)**
Proteobacteria	17.27
Actinobacteria	14.4
Acidobacteria	13.92
Chloroflexi	8.09
Verrucomicrobia	7.51
Bacteroidetes	6.68
Planctomycetes	5.98

Group Wet	
**Species**	**Contribution (%)**
Proteobacteria	17.86
Actinobacteria	17.04
Acidobacteria	12.98
Chloroflexi	7.97
Verrucomicrobia	6.68
Bacteroidetes	6.49
Thaumarchaeota	5.88

Groups Dry & Mesic	
**Species**	**Contribution (%)**
Actinobacteria	9.23
Acidobacteria	9.01
Proteobacteria	8.69
Verrucomicrobia	7.6
Bacteroidetes	6.86
Thaumarchaeota	6.73
Chloroflexi	5.98
Patescibacteria	5.57
Firmicutes	5.23
Planctomycetes	4.53
Armatimonadetes	3.68

Groups Dry & Wet	
**Species**	**Contribution (%)**
Actinobacteria	10.06
Proteobacteria	9.81
Acidobacteria	9.45
Bacteroidetes	7.98
Thaumarchaeota	6.88
Chloroflexi	5.9
Verrucomicrobia	5.56
Firmicutes	5.35
Patescibacteria	4.23
Planctomycetes	4.18
Gemmatimonadetes	3.57

Groups Mesic & Wet	
**Species**	**Contribution (%)**
Actinobacteria	11.81
Proteobacteria	7.85
Acidobacteria	7.81
Verrucomicrobia	7
Thaumarchaeota	6.76
Patescibacteria	6.19
Bacteroidetes	5.74
Firmicutes	5.49
Chloroflexi	5.07
Planctomycetes	4.6
Latescibacteria	2.93

Post-hoc SIMPER Analysis (fungi)

Group Dry (Phylum level)	
**Species**	**Contribution (%)**
Ascomycota	45.61
Basidiomycota	36.24

Group Mesic (Phylum level)	
**Species**	**Contribution (%)**
Ascomycota	41.93
Basidiomycota	34.36

Group Wet (Phylum level)	
**Species**	**Contribution (%)**
Ascomycota	47.49
Basidiomycota	33.43

Group Dry (Genus level)	
**Species**	**Contribution (%)**
Phallus	9.93
Cladosporium	5.82

Group Mesic (Genus level)	
**Species**	**Contribution (%)**
Phallus	10.31
Cladosporium	4.59

Group Wet (Genus level)	
**Species**	**Contribution (%)**
Phallus	9.47
Cladosporium	6.49

Relative Abundance (bacteria)

Phylum	Relative Abundance					
	Dry	Standard Error	Mesic	Standard Error	Wet	Standard Error
Actinobacteria	0.24	0.01	0.2	0.01	0.27	0.01
Proteobacteria	0.34	0.01	0.28	0.01	0.28	0.01
Acidobacteria	0.14	0.01	0.18	0.006	0.16	0.008
Verrucomicrobia	0.04	0.003	0.06	0.004	0.04	0.002
Bacteroidetes	0.05	0.007	0.04	0.002	0.04	0.005
Thaumarchaeota	0.02	0.002	0.03	0.003	0.04	0.003
Chloroflexi	0.05	0.003	0.06	0.003	0.06	0.003
Firmicutes	0.01	0.002	0.02	0.002	0.02	0.001
Patescibacteria	0.01	0.001	0.02	0.003	0.01	0.001
Planctomycetes	0.02	0.001	0.03	0.001	0.02	0.001
Armatimonadetes	0.006	0.0007	0.009	0.0008	0.006	0.0004
Gemmatimonadet	0.008	0.0007	0.011	0.0006	0.01	0.0007
Latescibacteria	0.001	0.0002	0.002	0.0002	0.001	0.0002
Cyanobacteria	0.0009	0.0003	0.001	0.0008	0.0003	9.45E-05
Rokubacteria	0.0018	0.0002	0.001	0.0002	0.002	0.0002
Entotheonellaeota	0.002	0.0002	0.002	0.0002	0.002	0.0002
Nitrospirae	0.0012	0.0001	0.001	0.0002	0.001	0.0002
BRC1	0.0007	9.49E-05	0.001	0.0001	0.0009	0.0001
Chlamydiae	0.0003	7.22E-05	0.0003	5.91E-05	0.0003	6.89E-05
Dependentiae	0.0001	3.87E-05	0.0002	5.51E-05	0.0002	5.75E-05
FBP	0.0002	4.38E-05	0.0001	3.51E-05	0.0003	9.16E-05
Elusimicrobia	9.73E-05	3.40E-05	0.0002	5.14E-05	0.0001	6.21E-05
Deinococcus-The	0.0001	4.02E-05	0.0001	3.65E-05	8.55E-05	2.17E-05
Fibrobacteres	4.92E-05	2.37E-05	0.0001	3.27E-05	0.00010481	3.23E-05
WS2	1.42E-05	1.42E-05	5.41E-05	3.14E-05	3.72E-05	2.14E-05

Supplementary Table S3: Genera that had differential relative abundance between dry, mesic and wet ecotypes.

Wet-Mesic

		Genus	log2FoldChange	baseMean	lfcSE	stat	pvalue	padj
Ecotype Predominance	Wet	Candidatus Berkiella	-53.69600475	4487.0305	3.5928627	-14.945187	1.67E-50	9.58E-48
		Cellvibrio	-49.37216613	3234.6123	3.592863	-13.741733	5.71E-43	8.16E-41
		Flavihumibacter	-49.24585783	3282.7311	3.592863	-13.706578	9.27E-43	1.06E-40
		Terrabacter	-47.1279787	65060.933	3.592862	-13.117114	2.63E-39	1.50E-37
		Parasegetibacter	-47.05867887	3846.2013	3.5928629	-13.097822	3.39E-39	1.76E-37
		Parviterribacter	-43.88974247	13494.783	3.5928622	-12.215816	2.56E-34	8.13E-33
		Cellulomonas	-40.78926288	63156.499	3.592862	-11.352861	7.18E-30	2.16E-28
		Solitalea	-35.98729472	5623.8688	3.5928626	-10.016329	1.29E-23	3.08E-22
		Sphaerisporangium	-35.77846377	4746.2196	3.5928627	-9.9582052	2.32E-23	5.11E-22
		Nonomuraea	-35.22712043	2243.5106	3.5928635	-9.8047477	1.07E-22	2.05E-21
		Achromobacter	-35.20242666	2852.6173	3.5928632	-9.7978757	1.15E-22	2.12E-21
		Acinetobacter	-34.99635569	1559.5749	3.5928642	-9.7405172	2.03E-22	3.62E-21
		Pseudorhodoferax	-34.80408778	7606.9705	3.5928624	-9.6870083	3.42E-22	5.76E-21
		Nocardia	-34.53241916	1078.8803	3.5928652	-9.6113873	7.16E-22	1.14E-20
		Leucobacter	-30.94650232	129303.61	3.592862	-8.613329	7.10E-18	8.49E-17
		Lacunisphaera	-30.64778369	11159.334	3.5928625	-8.5301856	1.46E-17	1.67E-16
		Nesterenkonia	-29.95405017	19716.071	3.5928623	-8.3370994	7.61E-17	8.06E-16
		Bosea	-29.94551614	94711.745	3.592862	-8.3347248	7.77E-17	8.08E-16
		Gemmatirosa	-29.70717817	106905.53	3.592862	-8.2683883	1.36E-16	1.36E-15
		Pelagibacterium	-29.49910818	40190.076	3.5928622	-8.2104759	2.20E-16	2.14E-15
		Aliihoeflea	-29.45844006	88564.373	3.592862	-8.199157	2.42E-16	2.31E-15
		Nocardiopsis	-28.60396592	10520.806	3.5928632	-7.961329	1.70E-15	1.52E-14
		Rubinisphaera	-28.3653283	13824.129	3.592863	-7.8949095	2.91E-15	2.56E-14
		Prosthecobacter	-28.28358096	11429.664	3.5928633	-7.8721562	3.49E-15	3.02E-14
		Xanthomonas	-27.10825799	70635.5	3.5928624	-7.545031	4.52E-14	3.80E-13
		Aureimonas	-26.76425252	134323.27	3.5928622	-7.4492844	9.38E-14	7.78E-13
		Rhodococcus	-9.15788243	698251.2	2.7412201	-3.3408052	0.0008354	0.0068261
		Pseudomonas	-2.160585255	74005051	0.772882	-2.7954919	0.0051821	0.0411687
	Mesic	Parafrigoribacterium	29.09180197	56398.574	3.5916551	8.0998318	5.50E-16	5.08E-15
		Ktedonobacter	29.68416311	42668.093	3.5916551	8.2647589	1.40E-16	1.38E-15
		Streptosporangium	32.26198109	237013.66	3.5336189	9.1300114	6.85E-20	9.33E-19
		Acidicapsa	32.43873919	13044.928	3.5916552	9.0316964	1.69E-19	2.25E-18
		Pseudoduganella	45.94451101	59743.599	3.591655	12.792017	1.82E-37	7.99E-36

Dry - Mesic

		Genus	log2FoldChange	baseMean	lfcSE	stat	pvalue	padj
Ecotype Predominance	Mesic	Microbispora	-28.59855246	4343.2651	3.6	-8.0	1.0E-15	9.7E-15
		Sorangium	-28.0929368	61271.589	3.6	-7.9	3.3E-15	3.0E-14
		Zavarzinella	-27.51211317	40054.456	3.6	-7.7	1.2E-14	1.1E-13
		Candidatus Udaeobacter	-0.526857546	22310063	0.2	-2.7	6.8E-03	4.8E-02
	Dry	Allorhizobium-Neorhizobium-Pararhizobium-Rhizobium	1.27136085	18804815	0.3	4.1	3.6E-05	2.8E-04
		Pseudomonas	2.869484026	74005051	0.8	3.7	1.8E-04	1.3E-03
		Cellulomonas	10.78926288	63156.499	3.6	3.0	2.6E-03	1.9E-02
		Rhodococcus	11.05824804	698251.2	2.7	4.1	4.8E-05	3.7E-04
		Parviterribacter	13.88974247	13494.783	3.6	3.9	1.1E-04	7.9E-04
		Parasegetibacter	17.05867887	3846.2013	3.6	4.8	1.9E-06	1.6E-05
		Flavihumibacter	19.24585783	3282.7311	3.6	5.4	7.9E-08	6.7E-07
		Cellvibrio	19.37216613	3234.6123	3.6	5.4	6.5E-08	5.6E-07
		Candidatus Berkiella	23.69600475	4487.0305	3.6	6.6	3.8E-11	3.4E-10

Dry-Wet

		Genus	log2FoldChange	baseMean	lfcSE	stat	pvalue	padj
Ecotype Pre	Dry	Allorhizobium-Neorhizobium-Pararhizobium-Rhizobium	1.697869148	18804815	0.3024529	5.6136648	1.98E-08	1.62E-07

Supplementary Table S4: Soil Total Carbon and Nitrogen measurements in the ecotypes.

Soil Total Carbon and Nitrogen ratio between the ecotypes

Sample#	sample name	Ecotype	Weight(mg	%Nitrogen	%Carbon	N mg/g	C mg/g	C:N
1	D_CDB_R1	Dry	51.378	0.12586616	1.27259493	1.2586616	12.725949	10.1107
2	D_CDB_R2	Dry	53.432	0.11613842	1.2064116	1.1613842	12.064116	10.387705
3	D_CDB_R4	Dry	53.081	0.1395624	1.35911012	1.395624	13.591101	9.7383689
4	D_CDB_R5	Dry	50.191	0.13225038	1.29099119	1.3225038	12.909912	9.7617198
5	D_CDB_R6	Dry	51.558	0.12399778	1.20246542	1.2399778	12.024654	9.6974755
6	D_CDB_R7	Dry	51.587	0.11520684	1.11824119	1.1520684	11.182412	9.7063787
7	D_CDB_R8	Dry	51.337	0.12515576	1.06057024	1.2515576	10.605702	8.4740025
8	D_CDB_R9	Dry	FAILED					
9	D_REL_R10	Dry	52.24	0.12766954	1.34224749	1.2766954	13.422475	10.513451
10	D_REL_R2	Dry	52.589	0.12082496	1.19634748	1.2082496	11.963475	9.9014926
11	D_REL_R3	Dry	53.044	0.18614948	2.60544086	1.8614948	26.054409	13.996498
12	D_REL_R4	Dry	52.103	0.16407254	1.85446179	1.6407254	18.544618	11.302694
13	D_REL_R6	Dry	52.806	0.10916174	0.97304106	1.0916174	9.7304106	8.9137553
14	D_REL_R7	Dry	53.048	0.19842988	2.74373603	1.9842988	27.43736	13.827232
15	D_REL_R8	Dry	51.272	0.13409081	1.26621735	1.3409081	12.662174	9.4429838
16	D_REL_R9	Dry	51.593	0.12833494	1.09087074	1.2833494	10.908707	8.5001851
17	D_SAL_R1	Dry	50.684	0.24322365	3.09371352	2.4322365	30.937135	12.719624
18	D_SAL_R2	Dry	53.079	0.13768235	1.60882509	1.3768235	16.088251	11.68505
19	D_SAL_R3	Dry	51.192	0.14293207	1.67194903	1.4293207	16.71949	11.697508
20	D_SAL_R4	Dry	53.486	0.18217057	2.06426001	1.8217057	20.6426	11.331468
21	D_SAL_R5	Dry	53.024	0.13299021	1.34117496	1.3299021	13.41175	10.084764
22	D_SAL_R6	Dry	52.06	0.14176103	1.54762185	1.4176103	15.476218	10.917117
23	D_SAL_R7	Dry	51.623	0.13406302	1.51145256	1.3406302	15.114526	11.274194
24	D_SAL_R8	Dry	51.861	0.13065507	1.14901936	1.3065507	11.490194	8.7942963
25	D_SAL_R9	Dry	51.768	0.13484165	1.21216655	1.3484165	12.121665	8.9895558
26	D_WEB_R1	Dry	51.785	0.16956137	2.22312522	1.6956137	22.231252	13.111036
27	D_WEB_R10	Dry	51.584	0.13331465	1.32243526	1.3331465	13.224353	9.9196541
28	D_WEB_R3	Dry	52.731	0.23939686	3.67224741	2.3939686	36.722474	15.339581
29	D_WEB_R5	Dry	52.668	0.14329161	1.35814404	1.4329161	13.58144	9.4781828
30	D_WEB_R6	Dry	52.077	0.14015719	1.45140004	1.4015719	14.514	10.355516
31	D_WEB_R7	Dry	51.471	0.13272299	1.50739717	1.3272299	15.073972	11.357469
32	D_WEB_R8	Dry	51.345	0.20947336	2.25137568	2.0947336	22.513757	10.74779
33	D_WEB_R9	Dry	51.399	0.12934557	1.08823371	1.2934557	10.882337	8.4133824
34	M_KON_R1	Mesic	51.856	0.28243446	4.836658	2.8243446	48.36658	17.124886
35	M_KON_R10	Mesic	51.002	0.15516752	1.69003797	1.5516752	16.90038	10.891699
36	M_KON_R2	Mesic	53.644	0.16247903	2.14086485	1.6247903	21.408648	13.176253
37	M_KON_R3	Mesic	52.106	0.12855211	1.39935088	1.2855211	13.993509	10.885476
38	M_KON_R4	Mesic	50.111	0.1580368	1.79208076	1.580368	17.920808	11.339642
39	M_KON_R5	Mesic	52.661	0.13476701	1.30328107	1.3476701	13.032811	9.6706238
40	M_KON_R6	Mesic	52.4	0.1540591	1.64094722	1.540591	16.409472	10.651414
41	M_KON_R7	Mesic	51.805	0.13297775	1.48742115	1.3297775	14.874212	11.185489
42	M_KON_R8	Mesic	51.49	0.13146809	1.23814893	1.3146809	12.381489	9.4178667
43	M_KON_R9	Mesic	50.434	0.12458076	1.06967592	1.2458076	10.696759	8.5862051
44	M_TAL_R1	Mesic	50.845	0.2147858	2.64841223	2.147858	26.484122	12.330481
45	M_TAL_R10	Mesic	51.438	0.12619856	1.20099878	1.2619856	12.009988	9.5167392
46	M_TAL_R2	Mesic	52.458	0.37952563	6.1284709	3.7952563	61.284709	16.147713
47	M_TAL_R3	Mesic	51.374	0.15727013	2.00421119	1.5727013	20.042112	12.74375
48	M_TAL_R4	Mesic	52.753	0.16826281	1.85117221	1.6826281	18.511722	11.001672
49	M_TAL_R5	Mesic	52.506	0.14054048	1.64043844	1.4054048	16.404384	11.672355
50	M_TAL_R6	Mesic	50.444	0.12511635	1.31957376	1.2511635	13.195738	10.546773
51	M_TAL_R7	Mesic	51.209	0.11069865	1.16868961	1.1069865	11.686896	10.557397
52	M_TAL_R8	Mesic	51.33	0.11947325	1.19872582	1.1947325	11.987258	10.033424
53	M_TAL_R9	Mesic	51.236	0.12928601	1.1101625	1.2928601	11.101625	8.5868729
54	M_TOW_R1	Mesic	51.703	0.14169513	1.33248353	1.4169513	13.324835	9.4038769
55	M_TOW_R10	Mesic	51.346	0.1451395	1.48924601	1.451395	14.89246	10.260791
56	M_TOW_R2	Mesic	51.518	0.14368774	1.64641964	1.4368774	16.464196	11.458317
57	M_TOW_R3	Mesic	53.109	0.15083756	1.71427202	1.5083756	17.14272	11.365021
58	M_TOW_R4	Mesic	51.703	0.20629528	2.74034262	2.0629528	27.403426	13.283593
59	M_TOW_R5	Mesic	50.618	0.11819934	1.13110137	1.1819934	11.311014	9.5694389
60	M_TOW_R6	Mesic	52.166	0.1251142	1.35638654	1.251142	13.563865	10.841188
61	M_TOW_R7	Mesic	50.931	0.11518351	1.17267013	1.1518351	11.726701	10.180886
62	M_TOW_R8	Mesic	51.335	0.1394742	1.21681321	1.394742	12.168132	8.724289
63	M_TOW_R9	Mesic	51.578	0.15951844	1.62491655	1.5951844	16.249166	10.186387
64	W_12MI_R1	Wet	51.202	0.12747483	1.38699687	1.2747483	13.869969	10.880555
65	W_12MI_R10	Wet	50.193	0.13041882	1.30808914	1.3041882	13.080891	10.029911
66	W_12MI_R2	Wet	52.487	0.11728481	1.31636143	1.1728481	13.163614	11.223631
67	W_12MI_R3	Wet	52.676	0.14774492	1.88887608	1.4774492	18.888761	12.784711
68	W_12MI_R6	Wet	51.183	0.18891759	2.26864338	1.8891759	22.686434	12.00864
69	W_12MI_R7	Wet	54.291	0.1150469	1.19273901	1.150469	11.92739	10.367416
70	W_12MI_R8	Wet	50.969	0.1345278	1.16275811	1.345278	11.627581	8.6432551
71	W_DES_R10	Wet	51.946	0.13897274	1.50294292	1.3897274	15.029429	10.81466
72	W_DES_R3	Wet	52.372	0.1208384	1.18420255	1.208384	11.842026	9.7998864
73	W_DES_R5	Wet	50.686	0.14047831	1.38714409	1.4047831	13.871441	9.8744358
74	W_DES_R6	Wet	50.985	0.2093717	2.71959352	2.093717	27.195935	12.989308
75	W_DES_R7	Wet	FAILED					
76	W_DES_R8	Wet	50.931	0.18827426	2.31461692	1.8827426	23.146169	12.293857
77	W_DES_R9	Wet	51.004	0.13840945	1.29330552	1.3840945	12.933055	9.344055
78	W_FUL_R1	Wet	51.657	0.12209338	1.22309899	1.2209338	12.23099	10.017734
79	W_FUL_R10	Wet	51.468	0.12316339	1.22135949	1.2316339	12.213595	9.9165793
80	W_FUL_R2	Wet	50.567	0.1832488	2.32320929	1.832488	23.232093	12.677896
81	W_FUL_R3	Wet	51.98	0.14151388	1.58170092	1.4151388	15.817009	11.177002
82	W_FUL_R4	Wet	52.805	0.1302103	1.36049032	1.302103	13.604903	10.448408
83	W_FUL_R5	Wet	51.28	0.70322156	4.84333801	7.0322156	48.43338	6.8873571
84	W_FUL_R6	Wet	51.21	0.10900453	0.96836644	1.0900453	9.6836644	8.8837262
85	W_FUL_R7	Wet	52.883	0.10892857	1.13560903	1.0892857	11.35609	10.425263
86	W_FUL_R9	Wet	51.176	0.14371386	1.4126575	1.4371386	14.126575	9.8296537
87	W_WAL_R1	Wet	FAILED					
88	W_WAL_R10	Wet	52.719	0.12535134	1.2419759	1.2535134	12.419759	9.9079588
89	W_WAL_R2	Wet	51.629	0.12409785	1.40400565	1.2409785	14.040056	11.313699
90	W_WAL_R3	Wet	52.693	0.14020091	1.79990089	1.4020091	17.999009	12.838011
91	W_WAL_R4	Wet	52.121	0.13893215	1.54240036	1.3893215	15.424004	11.101824
92	W_WAL_R6	Wet	51.25	0.13123861	1.29171765	1.3123861	12.917176	9.8425125
93	W_WAL_R7	Wet	53.537	0.1166082	1.20557141	1.166082	12.055714	10.338651
94	W_WAL_R8	Wet	51.225	0.13362116	1.27967238	1.3362116	12.796724	9.5768696
95	W_WAL_R9	Wet	50.432	0.12632409	1.06906056	1.2632409	10.690606	8.4628402

Supplementary Figure S1: The top bacterial and archaeal taxa present in dry, mesic and wet ecotypes.

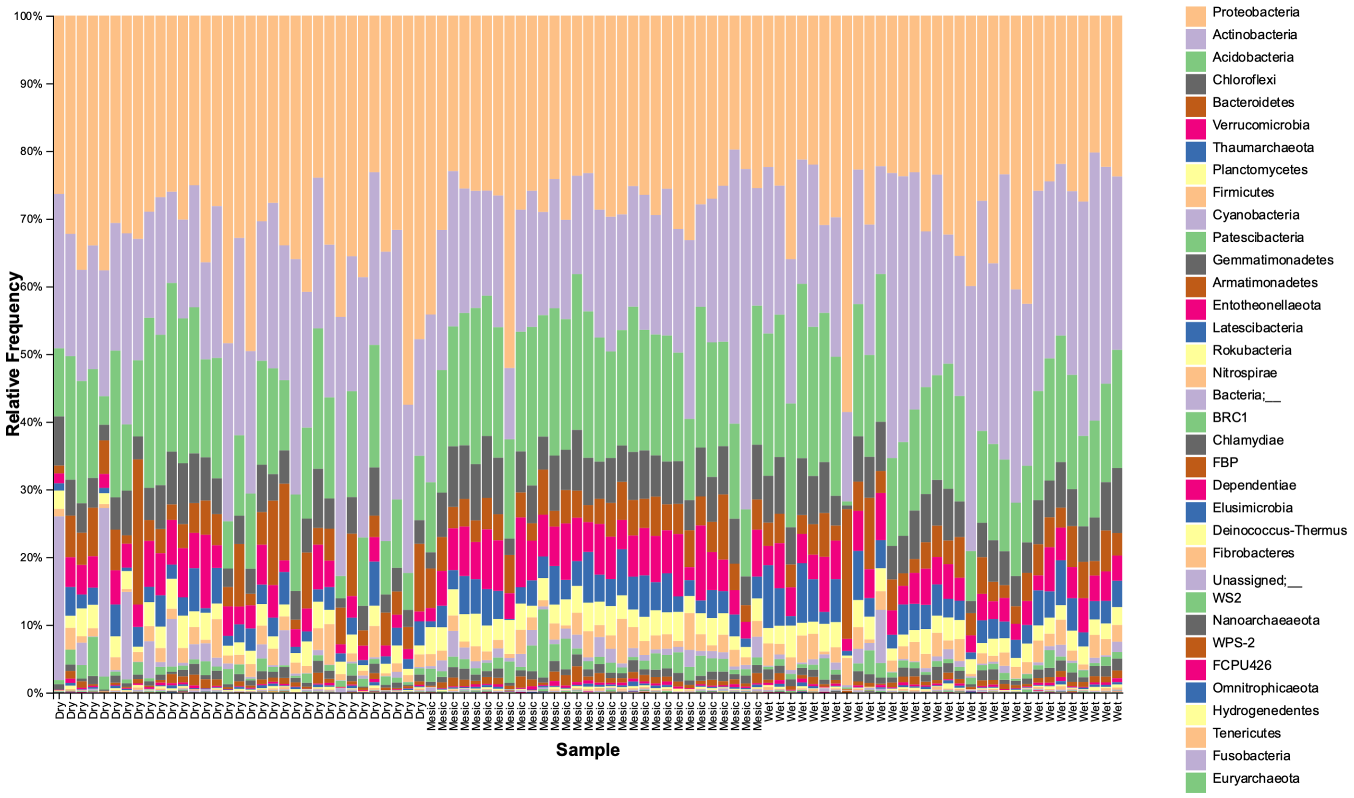
100%
90%
80%
70%
60%
50%
40%
30%
20%
10%
0%
Relative Frequency
Dry
Mesic
Wet
Sample
Proteobacteria
Actinobacteria
Acidobacteria
Chloroflexi
Bacteroidetes
Verrucomicrobia
Thaumarchaeota
Planctomycetes
Firmicutes
Cyanobacteria
Patescibacteria
Gemmatimonadetes
Armatimonadetes
Entotheonellaeota
Latescibacteria
Rokubacteria
Nitrospirae
Bacteria;__
BRC1
Chlamydiae
FBP
Dependentiae
Elusimicrobia
Deinococcus-Thermus
Fibrobacteres
Unassigned;__
WS2
Nanoarchaeaeota
WPS-2
FCPU426
Omnitrophicaeota
Hydrogenedentes
Tenericutes
Fusobacteria
Euryarchaeota

Supplementary Figure S2: The top fungal taxa present in dry, mesic and wet ecotypes.

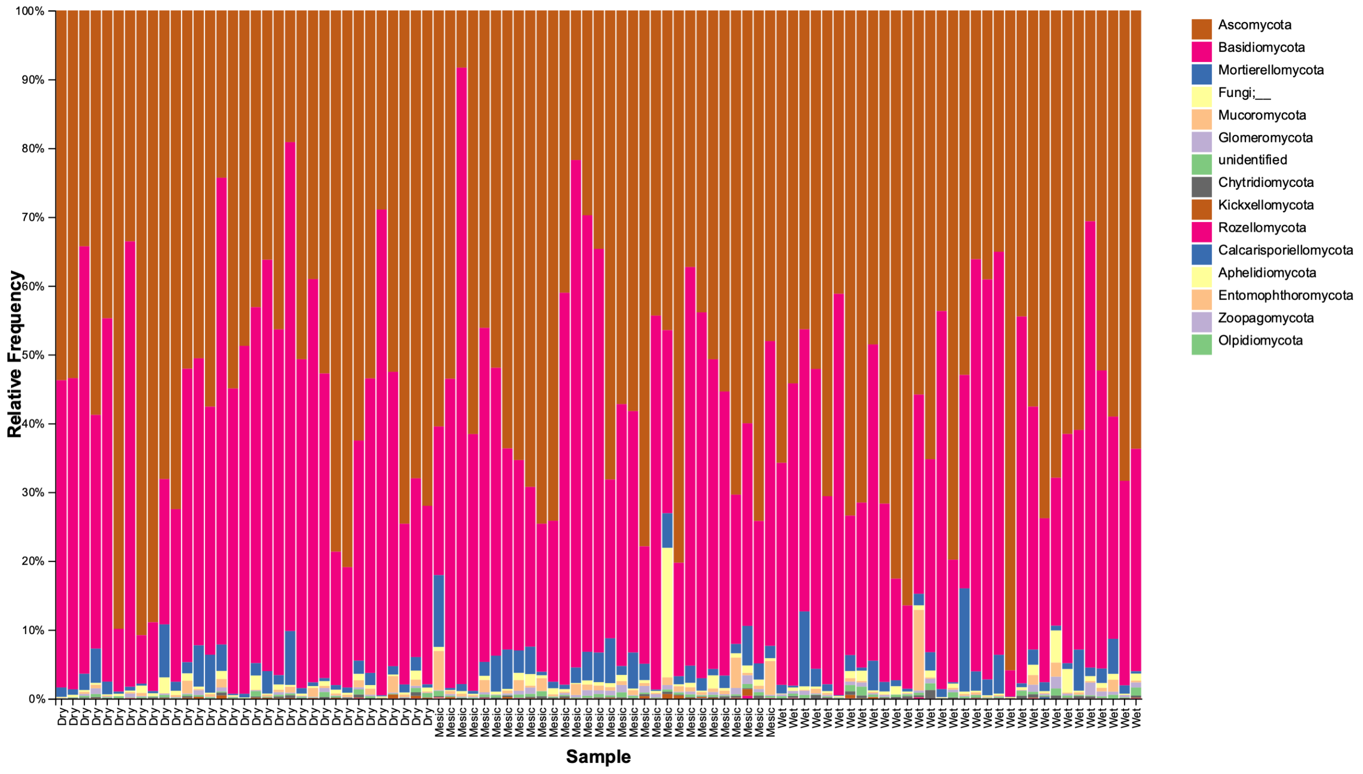

100%
90%
80%
70%
60%
50%
40%
30%
20%
10%
0%
Relative Frequency
Sample
Dry
Mesic
Wet
Ascomycota
Basidiomycota
Mortierellomycota
Fungi;__
Mucoromycota
Glomeromycota
unidentified
Chytridiomycota
Kickxellomycota
Rozellomycota
Calcarisporiellomycota
Aphelidiomycota
Entomophthoromycota
Zoopagomycota
Olpidiomycota
